# The Urine and Feces

**Published:** 1905-09

**Authors:** 


**The Urine and Feces.** A Practical Manual on the Urine and Feces in Diagnosis. By **Otto Hensel,** Ph.G., M.D., Bacteriologist to the German Hospital, New York, and Richard Weil, A.M., M.D., Pathologist to the German Hospital, New York, in collaboration with Smith Ely Jelliffe, M.D., Ph.D., Instructor in Pharmacology and Therapeutics, Columbia University, New York. Octavo, 334 pages, illustrated with 116 engravings and 10 colored plates. New York and Philadelphia: Lea Brothers & Co. 1905. (Cloth, $2.75 net.)

The output of works dealing with urinary analysis seems to have reached a climax in this book which is designed with a fixed purpose in view,—the production of a compact, handy and trustworthy guide to the study of the urine and the feces. The aim has been successfully accomplished and a book has been given the profession which leaves nothing to be desired and gives much to be thankful for. Combining all the good points of larger and bulkier works on analysis of urine and urinary diagnosis, it adds the feces—a heretofore much neglected field. The very excellent arrangement of the divisions of analytic work is welcome. There is an apparent and refreshing directness in the presentation of the normal and abnormal constituents and in the tests and findings. What strikes one as particularly useful is the page and a half devoted to urinary diagnosis. In this space is given all the information in a more comprehensive form, that other writers have struggled with in an entire chapter—and with less satisfaction.

The portion of the book devoted to the feces is equally satisfactory. Nothing is overlooked—no point which could possibly be of aid to the general practitioner is ignored. Taken from cover to cover there can be no hesitancy in according it unstinted praise,—and declaring it one of the best, most complete and most satisfactory book which could reach the table of the every day practitioner. He will find much good in even a cursory reading of it. N. W. W.

